# Do auditory brainstem implants favor the development of sensory integration and cognitive functions?

**DOI:** 10.1002/brb3.3637

**Published:** 2024-08-05

**Authors:** Banu BAŞ, Nuriye Yıldırım Gökay, Zehra Aydoğan, Esra Yücel

**Affiliations:** ^1^ Faculty of Health Sciences, Department of Audiology Ankara Yıldırım Beyazıt University Ankara Turkey; ^2^ Faculty of Health Sciences, Department of Audiology Gazi University Ankara Turkey; ^3^ Faculty of Health Sciences, Department of Audiology Ankara University Ankara Turkey; ^4^ Faculty of Health Sciences, Department of Audiology Hacettepe University Ankara Turkey

**Keywords:** auditory brainstem implants, cognitive, sensory integration

## Abstract

**Background:**

Information about the development of cognitive skills and the effect of sensory integration in children using auditory brainstem implants (ABIs) is still limited.

**Objective:**

This study primarily aims to investigate the relationship between sensory processing skills and attention and memory abilities in children with ABI, and secondarily aims to examine the effects of implant duration on sensory processing and cognitive skills in these children.

**Methods:**

The study included 25 children between the ages of 6 and 10 years (mean age: 14 girls and 11 boys) with inner ear and/or auditory nerve anomalies using auditory brainstem implants. Visual‐Aural Digit Span Test B, Marking Test, Dunn Sensory Profile Questionnaire were applied to all children.

**Results:**

The sensory processing skills of children are statistically significant and positive, and moderately related to their cognitive skills. As the duration of implant use increases, better attention and memory performances have been observed (*p* < .05).

**Conclusion:**

The study demonstrated the positive impact of sensory processing on the development of memory and attention skills in children with ABI. It will contribute to evaluating the effectiveness of attention, memory, and sensory integration skills, and aiding in the development of more effective educational strategies for these children.

## Graphical abstract

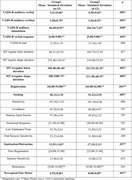

## 1 INTRODUCTION

Development is a dynamic process that reflects the rapid growth of interrelated functions in the cognitive, physical, and socio‐emotional areas. Cognitive abilities refer to a range of skills including language, memory, attention, reasoning, visualization, and perceptual function (Almomani et al., 2021). Cognitive development is a multidimensional process intertwined with the development of sensory systems. Challenges or limitations in the reception/perception of one sense can detrimentally impact children's overall cognitive growth. Relevantly here, hearing loss has been strongly linked to cognitive deficits. The majority of the population with congenital or early acquired hearing impairment has a similar cognitive developmental picture. The different pictures are not caused by the hearing impairment itself but by difficult language learning and the lack of a developed communication system. Sensory theories claim that early auditory deprivation changes neuronal networks across different brain areas, and this leads to serial effects on various functions. For example, lack of exposure to sounds during early development negatively impacts the cognitive abilities, speech and language skills, and social development of affected children (Kral et al., 2007, 2013). These theories are not sensory in the sense that it refers to sensory integration per se, but is related to the limited reception of speech features that must be received in order to be able to identify word meanings. Sensory integration and deafness are more connected by the so‐called cognitive theory in the field of language habilitation in deaf children, which states that due to congenital or early deafness, a series of cascading perceptual and cognitive consequences occurs (Conway et al., 2009). Differences in the development of deaf children compared to hearing children have been attributed to language learning difficulties, but there is also research showing that the brain may develop differently. Deafness has a direct and indirect impact on neurocognitive development. The brain development of these children can be viewed through the interrelationships of sensory and cognitive theory (Conway et al., 2009; Kral, 2007, 2019). The research idea of the current study originates from this idea. In particular, auditory deprivation has significant effects on children's neurocognitive development (de Giacomo et al., 2013). When children are deprived of hearing the speech sounds, they need to learn the language, language problems primarily emerge. The absence of fully verbal communication leads to socioemotional and other cognitive issues. In general, the auditory deficit experienced by children with hearing impairment can negatively affect the normal development of cognitive, psychomotor, and behavioral skills and can subsequently lead to neurodevelopmental changes (Peñeñory et al., 2018). Hearing assistive devices used in the rehabilitation of hearing loss can not only improve communication abilities but also positively affect cognitive and other functions (Almomani et al., 2021; Colletti, 2007; Pisoni et al., 2016). Cochlear implants (CI) can be used to intervene in hearing loss in people with severe to profound sensorineural hearing loss. However, CIs may be contraindicated in cases of anatomical malformation in the inner ear and/or auditory nerve. Auditory brainstem implantation (ABI) is a preferred intervention option in these cases. Thus, ABIs are placed in the cochlear nuclei in the brainstem, bypassing the inner ear (Yildirim Gökay & Yücel, 2024; Yıldırım Gökay et al., 2024). This study delves into the pivotal question of whether sensory integration plays a role in shaping cognitive skills among children with auditory brainstem implants (ABIs). Here, sensory integration refers to the process by which the brain organizes and interprets sensory information from the environment. It encompasses information from the senses such as touch, taste, smell, sight, and hearing, along with proprioception and vestibular input (Dunn, 1997). The study also examines the duration of implant use to evaluate the effect of early access to auditory stimulation on sensory integration and some cognitive skills.

Auditory brainstem implants (ABIs) are among hearing assistive devices that are used in patients experiencing severe or very severe hearing loss due to lack of a cochlea and/or a dysfunctional cochlear nerve (Sennaroglu et al., 2013). In individuals where the use of ABIs is recommended, auditory perception development might be constrained. Moreover, these children may experience additional neurological, psychological, and physical disorders, such as attention deficit hyperactivity disorder (ADHD). Fortunately, there is evidence that early use of assistive devices (e.g., ABI or cochlear implants) and their consistent use significantly impact the auditory, speech and language, cognitive, academic, and social development of children who receive these devices (Aslan et al., 2022). Studies have shown that early auditory implantation (cochlear implantation or auditory brainstem implantation) and its careful use in children have a significant effect on their auditory, language, and speech skills, cognitive skills, and academic and social development (Glaubitz et al., 2021; Hassanzadeh et al., 2021). Besides, sensory integration skills are also crucial for the development of these skills in children with ABIs. For example, to achieve a goal, a person should be able to make changes in his/her emotions and behaviors through self‐management (Ayres & Robbins, 2005). This process, which is under the management of the prefrontal cortex, is called self‐regulation. Functions such as attention, memory, planning, reasoning, mood regulation, and problem solving, which are among the executive functions, are in the self‐regulation process. For sensory integration skills to be full and complete, self‐regulation skills must function properly (Steinberg, 2010).

Functional sensory systems are needed to establish intraneuronal connections with cognitive areas. Sensory integration, our ability to interact with the world, depends on the ability of the cognitive system to consistently identify, use, and integrate a variety of sensory inputs (Ayres & Robbins, 2005). This process involves different steps in which the central nervous system recognizes, organizes, perceives, interprets, and responds appropriately to sensory information (Williamson & Anzalone, 2001). Model of Sensory Processing, known as Winnie Dunn Model, emphasizes how individuals process and respond to sensory information in their surroundings. To explain how individuals perceive and respond to sensory stimuli, which affect their behavior, emotions, and overall functioning, this model categorizes sensory processing into four patterns based on neurological thresholds and behavioral response including low registration, sensory seeking, sensory sensitivity, and sensory avoiding. For example, some individuals have high sensory thresholds, meaning they need a lot of stimulation to detect a stimulus and respond to it, while others have low sensory thresholds, and they respond even to small amounts of stimulation (Dunn, 1997). Deficits in the processing or integration of sensory input during periods of ongoing development can affect age‐appropriate memory, learning, attention, motor development, academic achievement, and social relationships (Bharadwaj et al., 2012; Dunn, 2001). A limited number of studies have indicated that sensory integration skills may be adversely affected in children with ABIs (Baş & Yücel, 2023; Ertugrul et al., 2021; Yildirim Gökay & Yücel, 2023).

This study aims to assess sustainable attention and memory skills in children with ABIs (Colletti et al., 2015; Colletti et al., 2005; Sennaroğlu et al., 2016; Colletti et al., 2009) and sensory integration abilities. Memory is the power to store what has been experienced and learned in the mind, a repertoire (Baddeley, 2007). According to Baddeley and Hitch's memory model, memory has three main components: the central executive, which acts as the control system and controls the flow of information from auxiliary systems; the phonological loop, which stores the verbal content in the stimulus; and the visual–spatial sketchpad, which stores the visual–spatial data (Atkinson & Shiffrin, 1968). This process involves encoding, storing, and retrieving incoming sensory stimuli. In Atkinson and Shiffrin's (1968) sensory memory model, incoming stimuli are transferred to short‐term memory (STM), which forms a crucial part of the system. STM not only transfers information to long‐term storage but also acts as a working memory, which is responsible for selecting processing strategies and manipulating information (Atkinson & Shiffrin, 1968). It has been reported that children with auditory implants exhibit weak performance in short‐term and working memory‐based skills (da Silva Lima et al., 2023; Köse et al., 2022; Pisoni et al., 2016). Furthermore, a weakness in memory skills has been observed in children with ABIs compared with their normally hearing or other auditory implanted peers (Herbert et al., 2023; Yildirim Gökay & Yücel, 2023).

Attention involves the choice of one or more than one possible stimulus or thought at a given moment (Cohen et al., 1993; Fawcett et al., 2015). It is controlled by the dynamic interactions of multiple neural systems. Specialized attention areas are located within the parietal and temporal lobe structures. In the literature, there are additional findings on the contribution of temporal lobe areas, which are central to auditory processing skills, to auditory attention (Berninger & Nagy, 2008; Karakaş, 2013; Koppitz, 1981). Similar to memory skills, weaknesses in sustained attention, selective attention, and attention‐related cortical responses have been identified in children with auditory implants (Edwards et al., 2006; Nicastri et al., 2023; Saksida et al., 2022; Schierholz et al., 2021). Despite the limited number of studies on children with ABIs, similar weaknesses have been observed (Colletti, 2007; Yildirim Gökay & Yücel, 2023).

Furthermore, according to Dunn's model, children who use ABIs are affected in their sensory processing skills, particularly in the aspects of inattention/distractibility (Baş & Yücel, 2023). However, to the best of the authors’ knowledge, while there are a limited number of studies on sensory processing in children using ABIs in the literature (Baş & Yücel, 2023), no study has yet investigated its effect on cognitive skills. Therefore, this study primarily aims to explore the positive effect of sensory processing skills assessed with the Dunn Sensory Profile questionnaire, specifically on cognitive abilities, such as attention and memory, in children with ABIs. It also investigates whether the duration of ABI use affects cognitive and sensory integration skills. This study may shed light on future studies evaluating how these skills affect children with ABIs also allows us to assess their quality of life and the effects on their daily activities.

## 2 MATERIALS AND METHODS

The study was found appropriate in terms of medical ethics by the Ankara Yıldırım Beyazıt University Non‐Interventional Clinical Research Ethics Committee. The children and their parents participating in the study were informed about the purpose and scope of the study, and written consent forms were obtained from both the children and their parents.

### 2.1 Participants

Twenty‐five children aged 6−10 years (14 girls and 11 boys) with inner ear and/or auditory nerve anomalies who used ABIs participated in the study. These children met the inclusion criteria and were grouped according to their mean value of duration of implant use. Group 1 consisted of 12 children with duration of 63.25 ± 5.20 months, and Group 2 comprised 13 children with duration of 76.38 ± 2.95 months (*p* < .001).

Children who had their first implantation before the age of 4 years, used bilateral implants regularly for at least 3 years after implant activation, had an auditory implant fitting and audiological follow‐up within the last 6 months, and had hearing thresholds with ABIs of 30−45 dB HL (within the speech field) at frequencies of 500−4000 Hz were included in the study. As the current assessment tools are applicable to children aged 6 and above, the participants in the study were selected from this specific age range. Since it is hypothesized that long‐term auditory deprivation could potentially affect cognitive and sensory scores (Kral, 2013), all of children received their implants before their fourth birthdays. All tests were conducted at the normal conversational level, with a defined threshold set to a minimum of 45−60 dB HL hearing thresholds. The etiologies of hearing loss in children, mostly comprising idiopathic hearing loss, include histories of anoxia, hyperbilirubinemia, consanguinity, prematurity and low birth weight. All children were diagnosed with hearing loss through newborn hearing screening and used hearing aids by the age of 6 months at the latest. In addition, all children diagnosed with hearing loss received regular auditory rehabilitation and used auditory communication methods. Children who were diagnosed with additional disabilities in the cognitive, psychological, motor, social, and mental areas who were not age appropriate, who were not cooperative with the tests, and who did not volunteer to participate in the study were excluded.

### 2.2 Assessment tools

The children were initially informed about the test instructions. A trial module was initially applied for each test. To avoid confounding variables, all tests were administered by a single researcher using live speech at a normal conversational level. The duration of the entire testing session was approximately 30 min, and no cooperation difficulties were observed in any child. The tests described below were used in this study because of their specificity, validity, and reliability in reflecting hearing‐related cognitive skills.

#### 2.2.1 Visual‐Aural Digit Span Test B (VADS‐B) form

This test was developed by Koppitz in 1977 to measure attention and/or short‐term memory. It is a neuropsychological test that comprehensively measures visual and auditory modalities and verbal and written responses, and it also provides a measure of sequencing ability (Koppitz, 1981). In the VADS‐B, the numbers are presented auditory or visually, and the responses are verbal or written. With this structure, VADS‐B measures sensation–response integration ability as intrasensory (auditory–verbal (AV), visual–written (VW)) and intersensory (auditory–written (AW), visual–verbal (VV)). It reflects the functions of the prefrontal cortex related to attention skills and the hippocampus because of its relationship with short‐term memory capacity (Karakaş, 2013). The administration of the test consists of four stages: AV, VV, AW, and VW (Berninger & Nagy, 2008). In the AV (Adam‐Darque et al., 2020) subtest, the sequences of numbers starting from a sequence of 3, one number per second, are read to the participants, and they are asked to repeat what they hear in order. In the VV subtest, the numbers are shown one by one from a booklet, and the participants are expected to say what they see in order. In the AW subtest, the children are asked to write the number sequences they hear on a piece of paper in order. In the VW (Berninger & Nagy, 2008) subtest, they are asked to write the number sequences shown in the booklet in order. The highest number of correctly repeated sequences is recorded as the score. This short‐term memory capacity (i.e., memory span) varies at 7 ± 2 units (Karakaş, 2013; Koppitz, 1981). The maximum score that can be obtained from each of the subtests is 9.

#### 2.2.2 Marking Test (MT)

This test was developed by Weintraub and Mesulam, and it measures visual and spatial scanning and/or perception skills, attention, reaction speed, and attack time (Karakaş, 2013). In MT performance, there is a sensory component related to perceptual errors, a motor component related to scanning and finding stimuli, and a motivational component, including affective features (Karakaş, 2013). It is a visual–spatial task involving sensory, motor, and affective components, as well as sustained attention. MT findings are considered to reflect the right cerebral hemisphere, especially parietal lobe functions (Lezak, 2004).

The MT consists of four test forms: regular letters, regular shapes, irregular letters, and irregular shapes. Each MT form contains 60 target stimuli, 15 in each quarter of the page. The individual is expected to encircle the target letter or shape as soon as possible without any errors. Every time the individual marks 10 letters or shapes, the tester gives a different color pen, and the marking continues with the new color. The purpose of this is to map the direction and flow of visual–spatial scanning. The best score for each form is 60. The completion times are compared with the norm values according to age. The administration time of the test takes approximately 20 min (Karakaş, 2013). In this study, the scanning time score was analyzed based on the MT findings.

#### 2.2.3 Dunn sensory profile questionnaire

This test was developed by Winnie Dunn in 1999 to measure sensory processing skills and to determine their effect on the child's daily life. The Sensory Profile Questionnaire, whose Turkish validity and reliability were confirmed by Kayıhan et al. (2015), is a standardized method for evaluating a child's functions in a wide range. It consists of 125 questions describing children's responses to sensory input. The questions are grouped into the sensory processing section, modulation section, and behavioral and emotional responses section. A five‐point Likert scale is used, which is filled in according to the frequency of these responses. As a result of the evaluation of the questionnaire, the raw scores are given verbal categorical equivalents, such as definite difference, probable difference, and typical performance. The questionnaire is completed by the parent or primary caregiver (Dunn, 1997; Dunn & Daniels, 2002).

### 2.3 Statistical analysis

Statistical analysis was performed using the SPSS (statistical package for the social sciences) version 26.0 software (IBM Corp.; Armonk, NY, USA). The sample size was determined using the G*Power version 3.1 software (with the parameters: correlation of interest ρH1 = 0.5, α error rate = 0.05, power = 0.85). Whether the data fit the normal distribution was evaluated with histogram curves and bell curves. Percentage values for categories variables such as gender were used for the descriptive statistics. The mean ± standard deviation values were used in the descriptive statistics of age variable. Twenty‐five children with ABI satisfying the inclusion criteria were grouped according to the duration of implant use. Group 1 consisted of 13 children with an implant usage period of 60–75 months and Group 2 consisted of 12 children with a usage period of 75–90 months. The values of VADS‐B and MT were compared between groups. Student's *t*‐test or Mann–Whitney *U* test was used for two‐group comparisons. In addition, the correlation between sensory profile and VADS‐B and the correlation between sensory profile and Marking tests were examined. A *p*‐value of .05 was considered statistically significant.

## 3 RESULTS

The mean age of the children in the Group 1 (7 female, 5 male) was 104.16 months (SD = 7.42), and it was 99.61 months (SD = 7.90) in the Group 2 (6 female, 7 male). There was no statistically significant difference between the two groups in terms of chronological age (*p* = .136). All children were diagnosed with hearing loss before 6 months and started using bilateral hearing aids. Table 1 presents the demographic information of children. Accordingly, there was a statistically significant difference between the groups in terms of duration of implant use (*p* < .001).

**TABLE 1:** Demographic characteristics (*N* = 25).

	Group 1	Group 2	
	Mean ± standard deviation	Mean ± standard deviation	*p*
**Age of hearing loss diagnosis (months)**	4.25 ± 1.35	5.00 ± 1.08	.138
**Onset age of hearing aid (months)**	5.50 ± 0.90	5.46 ± 0.87	.915
**Age of first implantation (months)**	18.81 ± 2.78	19.00 ± 3.31	.887
**Age of initial aural rehabilitation (months)**	6.00 ± 1.50	5.90 ± 0.97	.850
**Duration of implantation (months)**	63.25 ± 5.20	76.38 ± 2.95	<.001
**Gender**	7 females (58%) 5 males (42%)	6 females (46%) 7 males (54%)	

*Note*: *p* < .05 is statistically significant.

The scores of Groups 2, which had been using the implant for a longer time, were higher in the AV (*p* = .002), AW (*p* = .003), auditory stimulation (*p* = .048), and verbal response (*p* = .045) categories of the VADS‐B test and a statistically significant difference was found between the groups. According to the sensory profile, Group 2 had higher scores and better sensory processing skills in registration (*p* = .043), seeking (*p* = .001), inattention/distraction (*p* = .037), and perceptual fine motor (*p* = .047), and a statistically significant difference was found between the groups. In the marking test, Group 1 completed the irregular letter (*p* = .003) and irregular shape (*p* = .009) steps in a much longer time and a statistically significant difference was found between the groups (Table 2).

**TABLE 2:** Comparison of VADS‐B, MT, and DP according to duration of implant use.

	Group 1 Mean/standard deviation (*n* = 13)	Group 2 Mean/standard deviation (*n* = 12)	*p*
**VADS‐B auditory‐verbal**	3.41 ± 0.66*	4.30 ± 0.63*	.002*
**VADS‐B auditory‐written**	2.58 ± 0.79*	3.46 ± 0.51*	.003*
**VADS‐B auditory stimulation**	96.69 ± 8.93*	104.16 ± 7.42*	.048*
**VADS‐B verbal response**	[4.00–9.00]**	[5.00–9.00]**	.045*
VADS‐B total	12.03 ± 1.54	11.16 ± 1.85	.066
MT regular letter duration	86.21 ± 23.91	109.75 ± 27.85	.057
MT regular shape duration	252.86 ± 105.87	218.08 ± 55.03	.066
**MT irregular letter duration**	309.00 ± 89.40*	221.53 ± 36.25*	.003*
**MT irregular shape duration**	288.2580.75*	211.38 ± 40.35*	.009*
**Registration**	[46.00–54.00]**	[44.00–62.00]**	.043**
**Seeking**	**90.33 ± 3.39**	**91.23 ± 5.59**	.001*
Sensitivity	101.92 ± 7.63	101.16 ± 6.46	.066
Avoidance	65.92 ± 4.46	66.08 ± 4.07	.787
Sensory input search	57.38 ± 3.94	59.83 ± 2.32	.787
Emotional response	[51.00–63.00]	[48.00–66.00]	.787
Low endurance/tone	33.76 ± 2.61	31.83 ± 3.53	.395
Oral sensory sensitivity	33.23 ± 4.06	31.08 ± 4.60	.100
**Inattention/distraction**	13.91 ± 1.03*	17.15 ± 2.11*	.037*
Poor registration	[24.00–31.00]	[23.00–31.00]	.787
Sensory sensitivity	13.46 ± 2.43	14.08 ± 2.35	.653
Motionless	[9.00–16.00]**	[8.00–16.00]**	.395
**Perceptual fine motor**	6.92 ± 0.86*	8.00 ± 0.85*	.047*

*Note*: *Independent *t*‐test, **Mann–Whitney *U* test, *p *< .05 is statistically significant. Bolded areas indicate that the statistical analysis is significantly different.

In the study group consisting of 25 children using ABI, the relationships between sensory processing and attention and memory skills were evaluated and shown in Tables 3 and 4. The lower the scores obtained by the children in the sensory profile evaluation, the greater the difference between them and their peers. There is a significant positive correlation between the sensory profile and the subparameters of VADS‐B (*p* < .05). There is a significant positive correlation between registration and auditory verbal, auditory written and general scores (respectively *r* = .356, *r* = .427, *r* = .355, and *p* < .005). There is a positive correlation between sensitivity and auditory stimulation; positive correlation between sensory input seeking and auditory stimulation and general score; positive correlation between inattention and auditory verbal, auditory written; positive correlation between perceptual fine motor and auditory written (Table 3).

**TABLE 3:** Correlation between sensory profile and VADS‐B.

		VADS‐B auditory‐verbal	VADS‐B auditory stimulation	VADS‐B auditory‐written	VADS‐B verbal response	VADS‐B total
**Registration**	***r*** ***p***	.356* **.037**	−178 .395	.427* **.040**	.099 .637	.355* **.039**
Seeking	***r*** ***p***	.115 .585	.020 .923	.211 .311	−.139 .506	.269 .193
Sensitivity	***r*** ***p***	.013 .951	.047 .824	−065 .758	−.117 .579	.086 .682
**Avoidance**	***r*** ***p***	.038 .856	.456* **.022**	.050 .811	−.283 .171	−.282 .172
**Sensory input search**	***r*** ***p***	.197 .346	.348* **.028**	.237 .254	−.287 .165	.362* **.036**
Emotional response	***r*** ***p***	−.104 .622	−.108 .608	.037 .860	.059 .781	−.021 .919
Low endurance/tone	***r*** ***p***	−.176 .401	−.127 .544	−.130 .536	−.185 .375	−.148 .480
Oral sensory sensitivity	***r*** ***p***	−.346 .090	−.008 .971	.010 .964	.091 .666	.039 .854
**Inattention/distraction**	***r*** ***p***	.460* **.021**	−.007 .973	.374* **.046**	.033 .877	.127 .545
Poor registration	***r*** ***p***	.088 .676	.098 .641	.183 .381	.125 .552	.038 .856
Sensory sensitivity	***r*** ***p***	.051 .807	.006 .976	.340 .097	.252 .224	−.014 .946
Motionless	***r*** ***p***	.168 .304	.094 .709	.214 .656	.079 .593	.000 .421
Perceptual fine motor	***r*** ***p***	.017 .936	−.048 .820	.382* **.037**	.010 .963	−.150 .475

*Note*: Spearman's/Pearson's correlation; *correlation is significant at the .05 level. Bolded areas indicate that the statistical analysis is significantly different.

**TABLE 4:** Correlation between sensory profile and marking test.

		MT regular letter duration	MT regular shape duration	MT irregular letter duration	MT irregular shape duration
Registration	***r*** ***p***	−.044 .836	−.098 .643	−.062 .769	−.100 .635
**Seeking**	***r*** ***p***	−.391* **.032**	−.439* **.038**	−.079 .707	−.339* **.024**
**Sensitivity**	***r*** ***p***	.464* **.020**	.079 .708	.076 .717	−.117 .579
Avoidance	***r*** ***p***	.261 .207	−.178 .396	.256 .217	−.014 .949
Sensory input search	***r*** ***p***	−.132 .531	−.153 .625	−.292 .156	−.088 .677
Emotional response	***r*** ***p***	.363 .074	.006 .977	.172 .412	.016 .940
Low endurance/tone	***r*** ***p***	.177 .397	.304 .140	.392 .053	.134 .523
Oral sensory sensitivity	***r*** ***p***	.192 .357	.080 .702	.297 .149	.140 .460
**Inattention/distraction**	***r*** ***p***	−.481* **.025**	−.308* **.034**	−.399* **.043**	−.408* **.048**
Poor registration	***r*** ***p***	.246 .235	−.048 .820	.368 .071	.177 .397
Sensory sensitivity	***r*** ***p***	−.041 .847	−.248 .232	.049 .815	.096 .647
Motionless	***r*** ***p***	.259 .212	−.154 .462	−.108 .606	.129 .537
Perceptual fine motor	***r*** ***p***	−.050 .813	.134 .523	−137 .512	.168 .423

*Note*: Spearman's/Pearson's correlation; *correlation is significant at the .05 level. Bolded areas indicate that the statistical analysis is significantly different.

Among the subparameters of sensory profile and MT, there is a negative correlation between seeking and MT regular letter duration, MT regular shape duration, MT regular shape duration, MT irregular shape duration; there is a negative correlation between inattention/attention deficit and MT regular shape duration, MT irregular shape duration, MT irregular letter duration (Table 4).

## 4 DISCUSSION

Auditory deprivation at an early age can have complex effects on the brain's remodeling and the processing abilities of other intact senses (Bharadwaj et al., 2012; Finney & Dobkins, 2001; Heming & Brown, 2005). The processing of environmental and body senses is important in the development of cognitive skills that are effective in all developmental areas of children. Children with severe/profound hearing loss have difficulty recording and using information because they cannot receive auditory stimuli with all their acoustic properties (Kutlu et al., 2021). This condition causes limitations in short‐term memory and working memory and affects children's language development and social and cognitive skills (Abraham et al., 2016; Yildirim Gökay & Yücel, 2023; Yıldırım Gökay & Yücel, 2021). Moreover, as children with hearing loss cannot fully receive auditory stimuli, they spend more effort listening and their attention span is shortened (Hicks & Tharpe, 2002). Thus, the cognitive skills and sensory processing skills of children with hearing loss should be supported, and these children should be included in rehabilitation programs.

Early initial auditory input is important because it provides plasticity and remodeling of the brain and supports sensory integration. Studies have shown that children with early initial and long‐term use of cochlear implants (CIs) have better language development and social and cognitive skills (Yıldırım Gökay & Yücel, 2021). In these studies, some of children using CIs caught up with their peers, those using ABIs showed a significant improvement in some cognitive parameters related to selective visual attention and multisensory executive functions over time, although they could not catch up with their peers (Almomani et al., 2021; Colletti, 2007). Moreover, it has been recommended that children with cochlear anomalies associated with cognitive impairments should not be deprived of ABI intervention (Colletti & Zoccante, 2008). Colletti (2007) evaluated the nonverbal cognitive skills and hearing performance of children using ABIs and found that improvement in auditory perception caused a significant improvement in cognitive parameters. Another study emphasized that children using ABIs needed more improvement in terms of memory and attention processes (Yücel et al., 2015).

When we grouped the participants in the study according to the duration of ABI use, the children who used implants for a shorter period of time had lower scores in auditory presentation verbal response, visual presentation verbal response, auditory presentation written response, and verbal response steps in the VADS‐B test. In this situation, the participants had more difficulty in auditory and verbal tasks in which hearing, and language skills intensified. On the contrary, when intersensory interaction was required, no difference was found in the different task combinations, especially in the visual and written substeps. It could be that children using ABIs have weaker auditory perception and develop visual skills because they mostly use lip reading and sign language (Yildirim Gökay & Yücel, 2024).

The difference between the two groups in terms of visual and spatial scanning and/or perception visual selectivity and visual–motor synchronization skills measured by the MT in terms of irregular letter and shape durations may be due to the weaker sensory integration skills of children who used ABIs for shorter period of time. In this task, the children were asked to mark the targets in an organized manner using fine motor skills in a form containing confusing stimuli along with the target shape or letter. Differences were found between the groups in sensory processing parameters in terms of registration, searching, and fine motor skills.

Sensory integration is a process that requires continuity. Proprioceptive, vestibular, and tactile systems constitute the basic senses of this integration in developmental stages (Ayres & Robbins, 2005). In sensory integration studies of children with hearing loss, the current situation has been linked to the degree of hearing loss of the child, the duration of amplification use, the duration of rehabilitation, and the presence of additional disabilities rather than the device used (Coulson‐Thaker, 2020; Veronese et al., 2023). According to a study in which children using ABI were compared with those using CIs, children using ABIs were weaker in the vestibular system, movement modulation, and inattention substeps in the sensory profiles and more successful in visual processing (Baş & Yücel, 2023). In the current study, the duration of use affected sensory integration, which is consistent with the literature. Children with short‐term use showed weaker performance in the Dunn sensory profile substeps of registration, inattention, and fine motor skills, while children in this group were more mobile and sensory seeking. The sensory profiles of children affect all stages of academic, social, and cognitive development as well as communication and learning skills (Dunn, 1997).

The registration parameters of the sensory profile are the perception of the sensory stimulus and the recording of the sensory stimulus (Dunn & Daniels, 2002). Children who fail to meet the norm values in this area do not react because they cannot perceive the stimuli and have difficulty fulfilling the tasks. When the relationship between the sensory profile and the VADS‐B, in which short‐term memory and working memory skills are evaluated, there is a positive correlation between registration and AV, AW, and general scoring. This may be due to the children's limitations, especially in perceiving and recording auditory stimuli. Sensory input seeking refers to the need for different sensory stimuli to regulate children's senses, ensure concentration, and control behavior (Ayres & Robbins, 2005). We find that the positive correlation between sensory seeking and auditory stimulation and overall scores is due to children's regulation skills, low concentration, and high listening effort. It has been shown that inattention in the sensory profiles of children using ABIs may be related to difficulties in sensory seeking, registration, and listening skills. There is a negative correlation between regular shape and letter duration and between irregular shape and letter duration, which are evaluated in the MT findings that specifically measure selective attention and sustained attention skills and search and inattention/distraction from the sensory profile substeps. To provide sensory and emotional regulation, to express themselves at the point of communication, or to attract attention by showing themselves, individuals behave very actively, show that they are in a sensory search, and have difficulty recording the sensations they obtain. Although it cannot be generalized with the current sample size, it is considered that their concentration time is short, and the time required to complete the test is longer due to their sensory search and recording limitations.

The limitations in attention and memory skills and the behaviors and attitudes of children using ABIs may be confused with conditions such as attention deficit hyperactivity disorder and autism. Another limitation of the study is the diverse brands of devices used by the children. All ABI‐using children who participated in the study had inner ear anomalies, but the inability to make a specific classification due to the small number of participants is one limitation of the study. The sample size should be expanded by considering etiologies. There is a need for a more rigorous methodological approach and further research on this topic. Although the applied tests yielded excellent results, definitive conclusions could not be drawn based solely on correlations. Confounding variables, such as the duration of ABI usage, age at hearing loss diagnosis, and the etiologies of hearing loss, need to be considered. Understanding the sensory sensitivity and reactions of a child is also important for the rehabilitation process.

## 5 CONCLUSIONS

This study demonstrated the importance of auditory and sensory processing in the development of cognitive and executive functions. The effects of sensory profiles and sensory processing skills on the rehabilitation of children with ABIs should be taken into consideration. In this process, a holistic and early evaluation is needed to prevent problems that children may experience. A multidisciplinary team should determine the unique needs and strengths of children and develop appropriate support and intervention strategies.

## AUTHOR CONTRIBUTIONS

**Banu BAŞ**: Software; methodology; conceptualization; resources; project administration; writing—original draft; writing—review and editing; investigation. **Nuriye Yıldırım Gökay**: Visualization; writing—review and editing. **Zehra Aydoğan**: Formal analysis. **Esra Yücel**: Supervision; conceptualization.

## FUNDING

The authors declare that they were not funded for this study.

## CONFLICT OF INTEREST STATEMENT

The authors have no conflict of interest to declare.

### PEER REVIEW

The peer review history for this article is available at https://publons.com/publon/10.1002/brb3.3637.

## Data Availability

All data generated or analyzed during this study are included in this article. Further enquiries can be directed to the corresponding author.
